# Usefulness of direct‐conversion flat‐panel detector system as a quality assurance tool for high‐dose‐rate ^192^Ir source

**DOI:** 10.1120/jacmp.v16i1.5068

**Published:** 2015-01-08

**Authors:** Yoshinori Miyahara, Hajime Kitagaki, Tomonori Nishimura, Kanae Itakura, Shinobu Takahashi, Masaki Yokokawa, Nobue Uchida, Taisuke Inomata

**Affiliations:** ^1^ Department of Radiology Shimane University Hospital Izumo Shimane Japan; ^2^ Graduate School of Medical Research Shimane University Izumo Shimane Japan; ^3^ Department of Radiology Shimane University Faculty of Medicine Izumo Shimane Japan; ^4^ Department of Radiation Oncology Kinki University Faculty of Medicine Osaka Japan; ^5^ Department of Radiation Oncology Tottori Prefectural Central Hospital Tottori Japan; ^6^ Department of Radiation Oncology Shimane University Faculty of Medicine Izumo Shimane Japan

**Keywords:** quality assurance, high‐dose‐rate brachytherapy, direct‐conversion flat‐panel detector

## Abstract

The routine quality assurance (QA) procedure for a high‐dose‐rate (HDR) ${}^{192}\text{Ir}$ radioactive source is an important task to provide appropriate brachytherapy. Traditionally, it has been difficult to obtain good quality images using the ${}^{192}\text{Ir}$ source due to irradiation from the high‐energy gamma rays. However, a direct‐conversion flat‐panel detector (d‐FPD) has made it possible to confirm the localization and configuration of the ${}^{192}\text{Ir}$ source. The purpose of the present study was to evaluate positional and temporal accuracy of the ${}^{192}\text{Ir}$ source using a d‐FPD system, and the usefulness of d‐FPD as a QA tool. As a weekly verification of source positional accuracy test, we obtained ${}^{192}\text{Ir}$ core imaging by single‐shot radiography for three different positions (1300/1400/1500 mm) of a check ruler. To acquire images for measurement of the ${}^{192}\text{Ir}$ source movement distance with varying interval steps (2.5/5.0/10.0 mm) and temporal accuracy, we used the high‐speed image acquisition technique and digital subtraction. For accuracy of the ${}^{192}\text{Ir}$ source dwell time, sequential images were obtained using various dwell times ranging from 0.5 to 30.0 sec, and the acquired number of image frames was assessed. Analysis of the data was performed using the measurement analysis function of the d‐FPD system. Although there were slight weekly variations in source positional accuracy, the measured positional errors were less than 1.0 mm. For source temporal accuracy, the temporal errors were less than 1.0%, and the correlation between acquired frames and programmed time showed excellent linearity ($R^{2}=1$). All ${}^{192}\text{Ir}$ core images were acquired clearly without image halation, and the data were obtained quantitatively. All data were successfully stored in the picture archiving and communication system (PACS) for time‐series analysis. The d‐FPD is considered useful as the QA tool for the ${}^{192}\text{Ir}$ source.

PACS number: 87.56.Fc

## I. INTRODUCTION

HDR brachytherapy using a ${}^{192}\text{Ir}$ radioactive source is a well‐established cancer treatment for interstitial and intracavitary tumors (e.g., esophagus, prostate, and uterine cervical cancer). In HDR brachytherapy, high radiation dose can be delivered to the target lesion, while limiting exposure to the surrounding normal tissue by adjusting both source position and dwell time.^1^, ^2^ Particularly at the point of precipitous dose gradient in the dose distribution, errors in the source positions and source dwell time may lead to inappropriate irradiation. Therefore, recommended QA^3^, ^4^, ^5^, ^6^, ^7^, ^8^ procedures for the ${}^{192}\text{Ir}$ source, such as positional and temporal accuracy, are important tasks for provide a high‐quality brachytherapy.

Currently, FPDs have been widely applied^(9)^ in the field of diagnosis and radiation therapy owing to improvements in imaging technology in the last decade. The d‐FPD has a photoconductor (amorphous selenium) which directly converts the incident photons into electrical charge. The advantage of the photoconductor is that the light scatter problem can be totally avoided, compared with the indirect‐conversion FPD and conventional X‐ray digital fluoroscopy system. The d‐FPD also has wide dynamic range and high spatial resolution,^(10)^ even when high energy photons are transmitted to the detector, providing sufficient imaging capability for the ${}^{192}\text{Ir}$ source.

The purpose of this study was to evaluate the ${}^{192}\text{Ir}$ source positional and temporal accuracy using a d‐FPD system, and its usefulness as a QA tool.

## II. MATERIALS AND METHODS

### A. Direct‐conversion FPD system

Specifications of the d‐FPD system (Shimadzu Co., Kyoto, Japan) are shown in Table 1. The d‐FPD is included in a digital radiography and fluoroscopy system, and provides high spatial resolution and a wide dynamic range.^11^, ^12^ The d‐FPD allows imaging with the ${}^{192}\text{Ir}$ source, even when high‐energy gamma rays are emitted to the detector. In addition, the d‐FPD system includes many useful image processing and measurement tools. For measurement of moved distances of the object, high‐speed image acquisition and real‐time smoothed mask (RSM) processing are available.^13^, ^14^ RSM processing is the software for digital subtraction radiography. Acquisition of mask images is not necessary for RSM, as arbitrary measurement of moved distance is possible by arbitrary frame setting to make a mask image from a series of obtained radiographic images. In case of remarkably short movements of an object, pixel‐shift manipulation of the image is possible to separate each overlapped object. These image processing methods allow measurement of movement distance on the high‐quality images, even though the target object is continuously moving.

**Table 1:** Specifications of the direct‐conversion FPD system

*FPD system*	*Digitex Safire (DAR9400‐SURE2.2)*
Detector type	Direct conversion
Detector material	Amorphous‐Selenium
Effective pixels	1472×1472
Field of view (FOV)	15.24, 22.86, 30.48, 38.10, 43.18 cm
Pixel pitch	150 μm
Dynamic range	14 bit
Frame rate	7.5 / 15 / 30 fps

### B. ${}^{192}\text{Ir}$ source and HDR remote afterloading system

**Figure 1:**
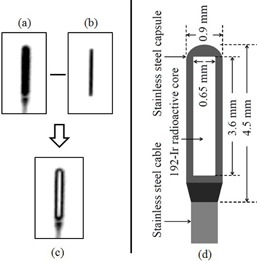
A d‐FPD low‐dose (a) (65 kVp, 200 mA, 20 msec) image of the ${}^{192}\text{Ir}$ source with the stainless steel cable. A high‐dose (b) (75 kVp, 280 mA, 28 msec) image of the ${}^{192}\text{Ir}$ core. A subtracted ((a)‐(b)) image (c). Schematic drawing (d) of the nucletron microSelectron v2 brachytherapy source.

Figure 1 shows the configuration of the ${}^{192}\text{Ir}$ source (microSelectron HDR v2; Nucletron B.V., Veenendaal, The Netherlands) and its d‐FPD images. Different radiographic doses provided varying depictions of the ${}^{192}\text{Ir}$ source. High energy (0.38 MeV) gamma rays were emitted from the ${}^{192}\text{Ir}$ radioactive solid core (Fig. 1(b)) which was enclosed in a stainless steel capsule and attached to a stainless steel cable (Fig. 1(a)). The cable was wound around the cable drum in the main unit of the microSelectron HDR. The ${}^{192}\text{Ir}$ source was controlled by stepper motors. The pulses from the shaft encoder of the stepping motor were counted when the irradiation source passed through the reference point (optical‐pair). The source was able to move at the speed of 50 cm per sec inside the catheter and applicator up to 1500 mm distance from the indexer. The source dwelling duration was controlled by electronic timer of the HDR afterloading system.^15^, ^16^ The half‐life of the ${}^{192}\text{Ir}$ source is 74 days, thus the source is generally exchanged every three months. Accuracy tests of source position and dwell duration are performed by operators (physicists) at the time of source exchange. The subsequent positional tests are performed every week before treatment. In addition, the calibration of the system (including source positional adjustment and source dwell time) is performed periodically.

### C. Measurement of the ${}^{192}\text{Ir}$ source position

**Figure 2:**
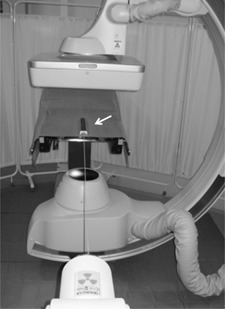
Overview of the source positional and temporal accuracy tests using d‐FPD and source position check ruler (arrow).

Source positional accuracy tests (Fig. 2) for three dwell points (1300, 1400, 1500 mm) were performed weekly using both d‐FPD and a source positional check ruler (Nucletron B.V.). In the manual setting of apparatus, the cable from the HDR body should be the height which is well aligned and exhibits good reproducibility. Because of short length of the source core, we selected 15.24 cm field of view (FOV) radiography for image magnification.

**Figure 3:**
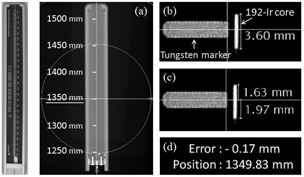
A photograph of the source position check ruler (left) and its d‐FPD images (30.48 cm FOV): (a) the radiographic tungsten markers were placed at 50 mm intervals in the check ruler; (b) definition of the reference length of long axis (3.6 mm) of the ${}^{192}\text{Ir}$ core; (c) measurement of the distance from the intersection between the midline of the tungsten marker and a ${}^{192}\text{Ir}$ core to both ends of ${}^{192}\text{Ir}$ core; (d) the ${}^{192}\text{Ir}$ source positional error was calculated as the difference from 1.8 mm.

Figure 3 shows the method of testing a source positional error using the check ruler (Fig. 3(a)) with radiographic tungsten markers. We obtained images of both tungsten markers and the ${}^{192}\text{Ir}$ core by single‐shot high‐dose radiography of each source dwell position, where the isocenter of the d‐FPD was set at the center of the targeted tungsten marker fixed at the dwell position in the check ruler. Parameters of all images were 75 kVp, 280 mA, 20 msec. For measurement of the source position, we used the digital scaling tool of the d‐FPD system.

In measurement of source positional errors, we first defined the reference length of the long axis (3.6 mm) of the ${}^{192}\text{Ir}$ core (Fig. 3(b)). After defining a reference length, finer measurement is possible by automatic pixel scale conversion from pixel (1472×1472 in whole displayed) to millimeter. We then measured the distance from the intersection between the midline of the tungsten marker and ${}^{192}\text{Ir}$ core to both ends of the ${}^{192}\text{Ir}$ core (Fig. 3(c)). Next, the difference from 1.8 mm was calculated as a positional error (Fig. 3(d)).

### D. Measurement of the ${}^{192}\text{Ir}$ source movement distance

**Figure 4:**
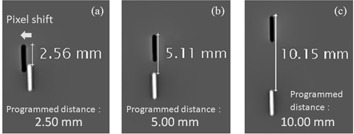
Measurement of source movement distance for three interval steps, 2.5 mm (left), 5.0 mm (middle), 10.0 mm (right). The ${}^{192}\text{Ir}$ source of premovement (white source) and postmovement (black source) were shown by digital subtraction method. In the case of 2.5 mm (a), the postmovement source was laterally pixel‐shifted by image processing to avoid overlapping of the sources.

Source movement distance was measured every week during a month for three types of interval steps (2.5, 5.0, 10.0 mm) of 11 dwell points, namely, ten sequential movements (Fig. 4). A series was measured in one day, and these series of source moving images were acquired by high‐speed image acquisition technique (15 frames per second (fps); temporal resolution of 0.07 sec) and RSM. All images were acquired by 70 kVp, 280 mA, 25 msec, and 30.48 cm FOV, and each source movement distance was measured by digital image subtraction method. In case of measurement for 2.5 mm source movement intervals, each postmovement image was laterally pixel‐shifted by image processing to avoid overlapping of the sources.

### E. Measurement of the ${}^{192}\text{Ir}$ source duration

The ${}^{192}\text{Ir}$ source temporal accuracy was measured by summing up the dwelled frames which were acquired by high‐speed image acquisition of 30 fps (i.e., temporal resolution) of 0.03 seconds (70 kVp, 280 mA, 25 msec, 30.48 cm FOV, 0.073 mm/pixel), and source duration varied from 0.5 to 30.0 sec. We evaluated the linearity between the acquired frames and programmed times. We also calculated the measured times as follows:

(1) Measured times=Dwelled frames×1/30

The errors between measured times and programmed times were calculated according to the following equation:

(2) Error(%)=100×(Measured times Programmed Times)/programmed times

## III. RESULTS & DISCUSSION

### A. Measurement of ${}^{192}\text{Ir}$ source position& movement distance

We measured positional errors from ${}^{192}\text{Ir}$ core images using the d‐FPD, as well as its scaling tool. In case of 15.24 cm FOV image analysis, the scaling tool was measurable up to the minimum 0.04 mm by pixel scale conversion. Exposure settings for all image acquisitions were defined to be the same. The visualization (image delineation) of the ${}^{192}\text{Ir}$ source core did not depend on the source strength.

**Figure 5:**
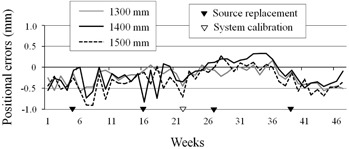
Source positional accuracy test. Weekly errors for three dwell positions are shown for one year.

**Figure 6:**
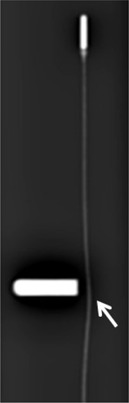
d‐FPD image of the skew cable (arrow).

The results of source positional accuracy tests for three dwell positions are shown in Fig. 5. Averages (± SD) of the three dwell positions were 1299.93±0.19, 1399.90±0.28, and 1499.71±0.36 mm, respectively. The acceptable ${}^{192}\text{Ir}$ source positional error as recommended by the AAPM (American Association of Physicists in Medicine) is within 1 mm.^(3)^ All measured source positions errors using the d‐FPD system in this evaluation were within ±1 mm. The weekly‐delineated ${}^{192}\text{Ir}$ source positions varied slightly from each of three programmed positions. Many factors are considered to have led to the positional differences, including frictional wear due to frequent use, equipment temperature differences, looseness of the source fixation in the tube, and cable skew (Fig. 6). The cable skew is infrequently observed. It may be caused by some slight pressure at the cable wound around the drum, or caused by friction between a source and the inner wall of tube. Although the cable skew is uncorrectable, the source positions errors were within the acceptable range.

The ${}^{192}\text{Ir}$ source movement is controlled by electronic pulses from the moment passing the reference optical‐pair to the programmed positions,^(16)^ and in the duration the source retracts to the base position. When the ${}^{192}\text{Ir}$ source is in‐drive, a small error at the starting point may result in a large error at the programmed position, and may also lead to the miscounting of electronic pulses as the source moves back and forth. Additionally, source positional calibration may be set slightly shorter to avoid system error occurring when moving beyond the maximum limit of 1500 mm. Thus, routine QA of the operational conditions of the ${}^{192}\text{Ir}$ source is important. The system is presumed to contain minor mechanical variance and error (approximately±0.5 mm); therefore, mechanical engineers are required to highly accurate source positional calibration. In addition, further studies are required for effectiveness of the source adjustment that collaborated with the d‐FPD image.

**Figure 7:**
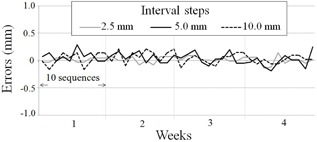
Source movement distance. Variation of errors in each source movement (ten sequences) distance for three interval steps, measured weekly for a month, is shown.

Figure 7 shows source movement distance measured weekly for a month. Series of images were acquired using high‐speed image acquisition with RSM, and each source movement distance was measured by digital subtraction method. Averages (± SD) of 11 dwell points for three interval steps were 2.49±0.04, 4.96±0.04, and 9.96±0.05 mm, respectively. All variations of error were less than 0.3 mm at all interval steps. In sequential source movements, the ${}^{192}\text{Ir}$ source simply moves forward constantly for a short distance after dwelling; thus, the error factor for sequential source movements may be less compared with the single‐shot measurement. Even for the shortest movement distance of 2.5 mm, the distance was successfully measured while avoiding overlapping of the source by lateral pixel shift (through image processing).

In this study, setting of apparatus and measurements were performed manually by an operator. We consider that measurement deviation between operators might be caused by the recognition variance of the ${}^{192}\text{Ir}$ source outline. In the examination using our d‐FPD scaling tool, the greatest interoperator deviation was 0.2 mm.

Various unique studies have been reported for QA of the ${}^{192}\text{Ir}$ source. Evans et al.^(17)^ reported source position quality assurance using two types of radiochromic film by the image coregistering technique. Kojima et al.^(18)^ measured source positions using a plastic scintillator CCD camera system. Jursinic^(19)^ uses a radiation diode in a unique test apparatus and a well chamber with an insert that provides a shield boundary. However, any research had difficulty in image resolution. We consider that the d‐FPD may improve the measurement accuracy due to its high resolution image and fine scaling.

### B. Measurement of ${}^{192}\text{Ir}$ source duration

**Figure 8:**
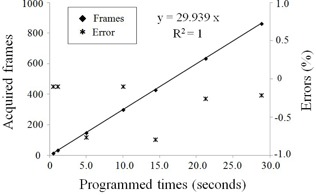
Source temporal accuracy test. Correlations are seen between the acquired frames, programmed times, and its errors.

Figure 8 shows the results of the source temporal accuracy test. Imaging frames for series of the ${}^{192}\text{Ir}$ source dwell motions were acquired while visually confirming from the beginning to the end of the motion. It was also possible to identify the ${}^{192}\text{Ir}$ dwell duration of only 0.5 sec by high‐speed image acquisition technique of 30 fps. Correlation between the programmed times and acquired frames showed excellent linearity ($R^{2}=1$). The recommended^3^, ^4^, ^6^ QA for the temporal accuracy does not require a stopwatch, but does require an electrometer that has been controlled treatment delivery duration and calibrated current source strength. The recommendation of the AAPM for ${}^{192}\text{Ir}$ source temporal accuracy deviation is within 1%.^(3)^ In our measurement using the d‐FPD system, all errors occurring in the measured source dwell duration were less than 1%. The d‐FPD enabled quantitative evaluation of source dwell time with series of ${}^{192}\text{Ir}$ source imaging frames using the high‐speed image acquisition. We consider that measurement of the temporal accuracy can be improved also in short duration time.

## IV. CONCLUSIONS

All measured data for QA of this afterloading system were within acceptable ranges recommended by Medical Physics societies. Source positional accuracy and temporal accuracy have been considered important to ensure the quality of brachytherapy. The d‐FPD has made it possible to clearly delineate a ${}^{192}\text{Ir}$ source. We have described an alternative method for QA using d‐FPD, characterized by its measurement by clear ${}^{192}\text{Ir}$ core imaging, quantitative evaluation, being filmless, and a simple evaluation method. Additionally, the acquired data can be stored, permitting time‐series analysis. Therefore, the d‐FPD system is considered to be a feasible quality assurance tool. The d‐FPD system may serve to improve aspects of QA, as well as the quality of HDR brachytherapy.
